# Book Reviews

**Published:** 1908-05

**Authors:** 


### BOOK REVIEWS

THE CARE OF THE BABY. By J. P. Crozier Griffith, M. D., Clinical Professor of Diseases of Children in the Hospital of the University of Pennsylvania. Fourth Revised Edition. 12mo of 455 pages, illustrated. Philadelphia and London: W. B. Saunders Company, 1907. Cloth, $1.50 net.

In this work, the author has endeavored to furnish a reliable guide for mothers anxious to inform themselves with regard to the best way of caring for their children in sickness and in health. He has made his statements plain and easily understood, in the hope that the volume may be of service not only to mothers and nurses, but also to medical students and to practitioners whose opportunities for observing children have been limited. The first chapter of the book discusses the hygiene of pregnancy, the method of calculating the date of confinement, and similar data. There are chapters on bathing, dressing, and feeding, on the hours for sleeping, and on physical and mental exercise and training.

A new fourth edition of this work has just been issued, bringing it right up to date. In it many additions and corrections have been made, and a large number of illustrations added. A second appendix has also been added, giving full information regarding the infants diet in health and disease, as well as some formulae.

We commend the work as a reliable and satisfactory guide for women and nurses.

---

DIAGNOSIS OF DISEASES OF CHILDREN. By LeGrand Kerr, M. D., Professor of Diseases of Children at the Brooklyn Postgraduate Medical School. Octavo of 542 pages, illustrated. Philadelphia and London: W. B. Saunders Company, 1907. Cloth, $5.00 net; Half Morocco, $6.50 net.

Dr. Kerr's work differs from all others on the diagnosis of diseases of children in that the objective symptoms are particularly emphasized. The author believes that as the objective symptoms are the main sources of information in diagnosing children's diseases the subject should be discussed with these symptoms as the foundation. The work is extremely practical, being written to help those who are engaged in the general practice of medicine to an early recognition of disease when it occurs in a child. The constant aim throughout has been to render a correct diagnosis as early in the course of the disease as possible, and for this reason differential diagnosis is presented from the very earliest symptoms. Just sufficient of etiology and pathology has been introduced to assist in arriving at right conclusions; and the sequelæ of the various diseases have been considered only to the extent that they may be anticipated and early recognized. The many original illustrations are of a higher order both from a practical and an artistic point of view. The physician will find these pictures a source of much information and help in his daily pediatric work.

---

THE TECHNIC OF OPERATIONS UPON THE INTESTINES AND STOMACH. By Alfred H. Gould, M. D., of Boston, Massachusetts. Octavo volume, containing 190 beautiful original illustrations, some of them in colors. Philadelphia and London: W. B. Saunders Company, 1906. Cloth, $5.00 net; Half Morocco, $6.00 net.

Dr. Gould's book is the direct result of the experimental study of gastro-intestinal technic, the operations having been first tried upon animals and then, in order to make the necesasry anatomic corrections, upon the cadaver. His idea is a new one, and will doubtless meet with immediate success. The object of the experiments was to simplify, where possible, the best gastro-intestinal operations, and the various steps and methods are shown by illustrations so clear, so anatomically accurate, and so pre-eminently practicable that but little text has been required. In fact, this was Dr. Gould's aim throughout: to present the technic of the standard operations upon the intestines and stomach by pictures alone, employing descriptive text only when it was impossible to express the idea pictorially. As the success of gastro-intestinal surgery depends upon an accurate knowledge of the elementary steps, a thorough account of repair is included, followed by a full description of all the important stitches, knots, and instruments used in intestinal surgery. The more important subjects treated are: the repair of intestinal wounds, including the structure of the intestines and the stomach, and experimental research on repair; suture materials, needles, tying knots, sutures, and clamps; the anatomy of the intestines, including intestinal localization; operations upon the intestines, operations upon the stomach. The beautiful illustrations, one hundred and ninety in number, are all original, and were made under Dr. Gould's direct supervision from actual operations.

---

### A PRACTICAL GUIDE TO THE EXAMINATION OF THE EAR.

---

BY SELDEN SPENCER, A. B., M. D., Instructor of Otology in Washington University, Aural Surgeon to Martha Parson's Free Hospital for Children. C. V. Mosby Medical Book and Publishing Company, St. Louis, Mo.

This little book is intended as a guide to undergraduate students in the course of Otology and as such well fills the aim of the author. Diagnosis is the fundamental part of any branch of medicine and many practitioners will be enabled, after a careful study of this brief work, to carry out their plan of diagnosis in a more systematic and intelligent manner.

---

### NERVOUS AND MENTAL DISEASES. FOR STUDENTS AND PRACTITIONERS.

---

BY CHARLES S. POTTS, M. D.

---

Professor of Neurology in the Medical-Chirurical College of Philadelphia. New (second) edition, thoroughly revised and greatly enlarged. In one 12mo. volume of 570 pages, with 133 engravings and 9 full-page plates. Price. cloth, $2.50 net. Lea & Febiger, Publishers, Philadelphia and New York.

The handling of nervous and mental diseases in a single volume offers manifest advantages to practitioners and students who wish a good grounding in two important subjects which have an obvious relationship. That Dr. Potts has accomplished this acceptably is indicated by the demand for repeated printings of his first edition, and now by the call for a revision. His book has always been noted for its clearness and evenness, the inclusion of everything to be expected in a manual, and the omission of recondite matters, which find their proper place in the large special works or in monographs. Dr. Potts carries his reader as far as most will care to go, qualifying him for examination or general practice on both subjects, and for their further pursuit in case he wishes to specialize. He has brought this new edition thoroughly abreast of the present day, incorporating all important advances and making many additions. The section on Mental Diseases has been completely rewritten to represent the radical change in the whole point of view from which this field is now regarded. As the book has grown larger by about one hundred pages in spite of condensation wherever possible, it may be said that the amount of information it contains has been increased in greater ratio than its pages, and the same is true of the illustrations. A number of colored plates have been introduced. In its new edition the book goes forward to fresh usefulness.

---

### PRACTICAL FEVER NURSING.

---

BY EDWARD C. REGISTER, M. D.

---

Professor of the Practice of Medicine in the North Carolina Medical College. Octavo volume of 352 pages, illustrated. Philadelphia and London: W. B. Saunders Company, Cloth, $2.50 net.

W. B. SAUNDERS COMPANY, Philadelphia and London.

This work completely covers the field of practical fever nursing. A nurse, before she can intelligently care for a fever patient, and anticipate all that is required of her by the physician, must have some knowledge of the disease and its medical treatment, some realization of the cause and significance of the signs and symptoms. Dr. Register, therefore, has described, in as non-technical a manner as possible, the pathology of the different fevers, their prognosis, and the various methods of treatment. The work is thoroughly practical and nurses will find it the *most valuable book on nursing yet published*. The illustrations show the nurse how to perform those measures that come within her province; such as bathing, hypodermoclysis, administration of antitoxin, pulse and temperature taking, etc.

---

### A MANUAL OF NORMAL HISTOLOGY AND ORGANOGRAPHY.

BY CHARLES HILL, PH. D., M. D.

Assistant Professor of History and Embryology, Northwestern University Medical School, Chicago. 12mo. volume of 463 pages, with 312 illustrations. Philadelphia and London: W. B. Saunders Company, 1906. Flexible leather, $2.00 net.

Dr. Hill's fifteen years' experience as a teacher of histology has enabled him to present a work characterized by clearness and brevity of style and a completeness of discussion rarely met in a book of its pretensions. Particular consideration is given the *mouth* and *teeth*. The subject of Organography is also included.

---

### MEDICAL GYNECOLOGY.

BY HOWARD A KELLY, A. B., M. D., L. L. D., T. R C. S. (HON. EDINB.)

Professor Gynecological Surgery in the Johns Hopkins University and Gynecologist to the Johns Hopkins Hospital, etc. D. Appleton and Company, New York.

Company, New York.

Dr. Kelly, with the assistance of a corps of able collaborators presents in a concise and interesting form the subject of Gynecology as it has evolved from its beginning as a minor specialty to its present dignity as a major surgical specialty.

Especial stress is justly placed upon the question of hygiene and prophylaxis which is one of the fundamental problems too frequently neglected. Drs. Lillian Welch and Mary Sherwood have written the chapter on the hygiene of growing girls. Dr. L. F. Barker is responsible for the chapter on neurasthenia, hyteria, and psychasthenia. This chapter is said to contain the first explicit and detailed statement made by Dr. Barker dealing with this class of patients. Other subjects are written by such men as Prince A. Morrow, E. J. Ill, T. R. Brown, and a number of others.

Two features make it of particular value to the medical student, first its concise form, second its "up-to-date" character.

The book is well bound, contains excellent illustrations, and is one we can heartily commend as a work of great value.

---

### SAUNDERS' FORTHCOMING BOOKS.

---

MESSRS. W. B. SAUNDERS COMPANY, medical publishers of Philadelphia and London, announce for publication before June 30th a list of books of unusual interest to the profession. We especially call the attention of our readers to the following:

Bandler's Medical Cynecology—Treating exclusively of the medical side of this subject.

Bonney's Tuberculosis.

Volume JI, Kelly and Noble's Gynecology and Abdominal Surgery.

olume IV, Keen's Surgery.

Grant's Constipation and Intestinal Obstruction.

Schamberg's Diseases of the Skin and the Eruptive Fevers.

John C. DaCosta, Jr.,'s Physical Diagnosis.

Todd's Clinical Diagnosis.

Camac's Epoch-Making Contributions in Medicine and Surgery.

All these works will be profusely illustrated with original pictures.

